# Unusual Presentation of Bilateral Radiation-Induced Angiosarcoma of the Breast

**DOI:** 10.1155/2020/5768438

**Published:** 2020-03-01

**Authors:** Umesh Jayarajah, Kavinda Nagodavithane, Oshan Basnayake, Sanjeewa Seneviratne

**Affiliations:** ^1^Professorial Surgical Unit, National Hospital of Sri Lanka, Colombo, Sri Lanka; ^2^Department of Surgery, Faculty of Medicine, University of Colombo, Sri Lanka

## Abstract

Radiation-induced sarcoma of the breast is an iatrogenic malignancy that occurs secondary to radiotherapy, which is most commonly given following breast conservation surgery. It has an incidence of 3.2 per 1,000 patients at 15 years and is associated with a poor prognosis. We report a 62-year-old female with a history of bilateral breast conservation surgery and radiotherapy 5 years ago presenting with bilateral angiosarcoma. This case report highlights the importance of considering radiation-induced angiosarcoma of the breast as a differential diagnosis in a patient with recurrent breast neoplasms. The challenges in the management with recent evidence on new treatment modalities are discussed.

## 1. Introduction

The management of breast cancer, the commonest cancer worldwide in women, has evolved during the last several decades where breast conservative treatment with systemic therapy has replaced radical surgery, improving quality of life and overall survival [1, 2]. However, this has resulted in an increased incidence of radiation-induced soft tissue sarcoma of the breast [3]. Radiation-associated angiosarcoma is the commonest histological variant and is associated with poor outcomes [3]. Following radiotherapy, the cumulative incidence of radiation induced sarcoma has been reported to be 3.2 per 1,000 patients at 15 years [3]. We report a 62-year-old female who presented with bilateral breast angiosarcoma 5 years after breast conservation surgery and radiotherapy. Furthermore, the challenges in the management with recent evidence on new treatment modalities are discussed.

## 2. Case Report

**Figure 1:**
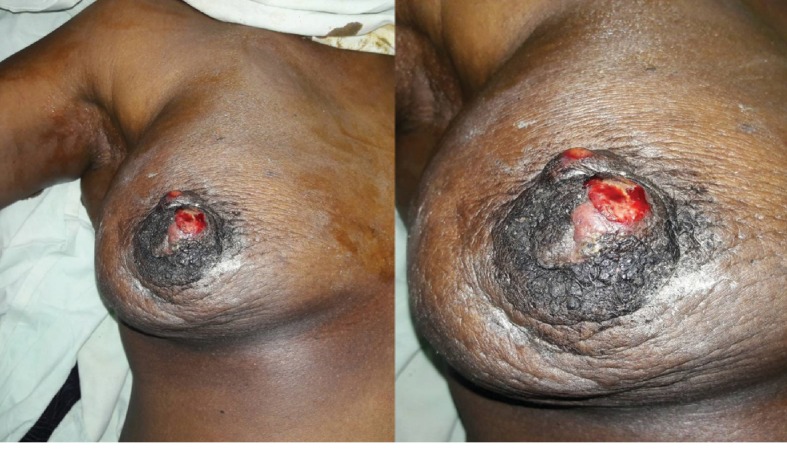
Fungating tumour with active bleeding from the erosion.

A 57-year-old Sri Lankan Sinhalese woman was diagnosed with bilateral stage IIB (T3N0M0) invasive ductal carcinoma, which was of Nottingham grade 2 and oestrogen receptor (ER), progesterone receptor (PR) positive, and human epidermal growth factor receptor-2 (HER-2) negative. She underwent bilateral wide local excision and level II axillary lymph node dissection with negative resection margins followed by standard bilateral adjuvant radiotherapy (50 Gy in 25 fractions with additional boosts of 5 Gy to each side using the linear accelerator) and 5 years of endocrine therapy with tamoxifen. Five years following the initial diagnosis, she presented with a rapidly enlarging right breast lump with skin erosion and bleeding of 2 weeks duration (Figure 1). Ultrasound scan of the breast showed a suspicious lesion and a core biopsy confirming the diagnosis of an angiosarcoma. She underwent right mastectomy after a multidisciplinary team discussion. Two weeks later, she presented with another small lesion of 0.5 cm size on the left breast skin. Excision biopsy of the lesion confirmed angiosarcoma and she underwent a left mastectomy.

**Figure 2:**
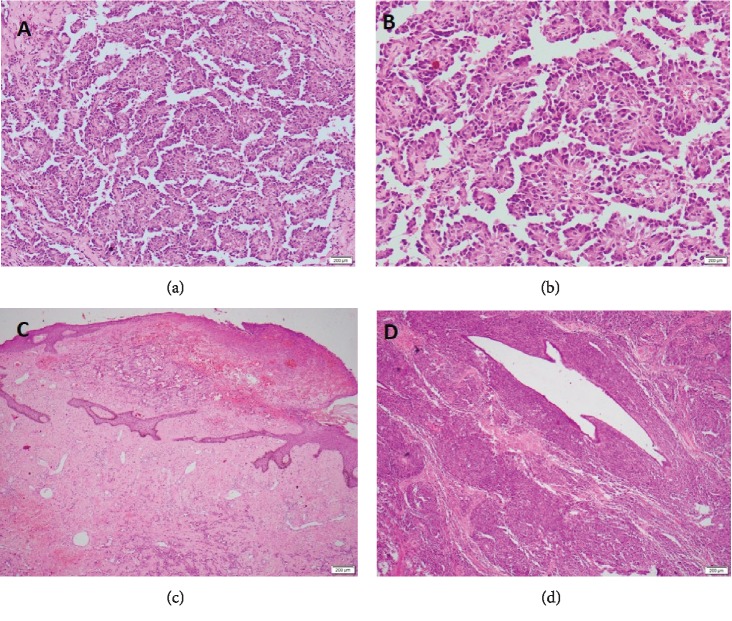
(a and b) Microscopic analysis showing anastomosing channels of vascular spaces lined by atypical cells with markedly pleomorphic vesicular nuclei and moderate eosinophillic cytoplasm. (c) Diffuse sheets and infiltrating cords of cells, areas of necrosis and blood lakes. (d) Tumour infiltration around the mammary ducts and surrounding fatty tissues.

Macroscopic analysis of the specimens showed solid, irregular, haemorrhagic lesions. The right-side lesion involved the nipple areolar complex with surface ulceration measuring 60 × 50 × 30 mm and another lesion was found in the central quadrant measuring 30 × 25 × 25 mm. Microscopic analysis of all lesions showed similar features with anastomosing channels of vascular spaces lined by atypical cells with markedly pleomorphic vesicular nuclei and moderate eosinophillic cytoplasm (Figure 2). Mitoses were frequent with atypical forms with a count of 23/10 high-power field. Diffuse sheets and infiltrating cords of cells, areas of necrosis, and blood lakes were noted. There was tumour infiltration around the mammary ducts and surrounding fatty tissues (Figure 2). The tumour grading was consistent with FNCLCC (Fédération Nationale des Centres de Lutte Contre Le Cancer) grade 2 with absent lymphovascular and perineural invasion. The resection margins were free of tumour. The left breast did not show any additional lesions.

**Figure 3:**
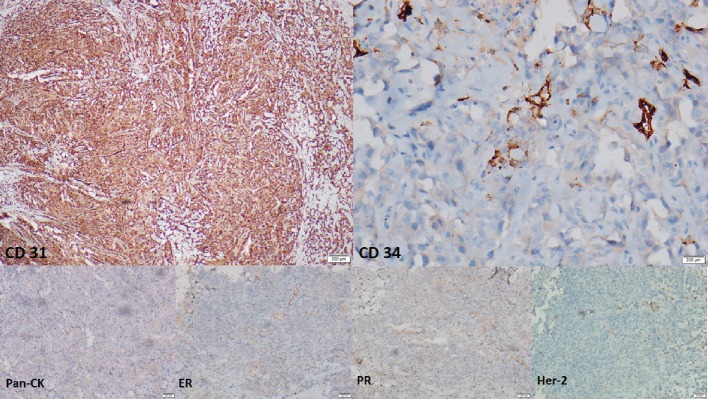
Immunohistochemical analyses showing strong and diffuse cytoplasmic and membrane positivity for CD 31 and occasional cytoplasmic and membrane positivity for CD 34. Tumour cells were negative for PanCK., ER, PR, and HER-2.

Immunohistochemical analyses showed strong and diffuse cytoplasmic and membrane positivity for CD 31 and occasional cytoplasmic and membrane positivity for CD 34 (Figure 3). Tumour cells were negative for pan cytokeratin (CK), ER, PR, and HER-2.

Contrast-enhanced computed tomography scan showed no evidence of metastatic disease. She was started on chemotherapy and was disease-free at 15 months follow up.

## 3. Discussion

In 1907, Borman et al. describes the first case of angiosarcoma of the breast, whereas the first case of radiation-induced secondary angiosarcoma was described Body et al. in 1987 [4]. With the evolution of breast conservativation surgery, there is increased usage of adjuvant local radiation which is significant risk factor for soft tissue sarcomas, particularly angiosarcoma [3, 5]. Due to its rarity, the evidence on radiation-induced angiosarcoma is restricted to case reports and series, and therefore, remains a challenge in terms of diagnosis and management [3, 5, 6].

The reported patient presented with a rapidly enlarging right breast lump with skin erosion and spontaneous bleeding, which is an unusual presentation. Several case reports describe skin discoloration, thickening, and dimpling as the clinical presentation which may be subtle, delaying the presentation and diagnosis [6, 7]. Lyou et al., described two cases which had multiple red papules and a palpable breast mass as the presenting feature [5]. Ashour et al. also described a painless lump and skin discolouration as presenting features. [4]. The presence of a palpable mass is a rare phenomenon, and our patient had a rapidly growing fungating mass with skin erosion and spontaneous bleeding which is unusual. Radiation induced sarcomas usually present after 10 years; however, it may present as late as 20 years following radiation [3]. The reported patient probably would have had and increased susceptibility as she developed bilateral disease within a relatively short time period of 5 years.

Management of radiation-induced angiosarcoma requires an aggressive surgical approach with complete resection with wide margins as an attempt for cure. Morgan et al. analysed 33 patients and found that a standard mastectomy may not be adequate [8]. The authors suggested that complete excision of all tissues exposed to radiation in contrast to isolated tumour resection may reduce tumour recurrence [8]. However, this approach may add to the surgical morbidity of the patient and still lacks data on long-term survival outcomes. Moreover, further radiation therapy has little benefit as most angiosarcomas are resistant to radiotherapy, and therefore, will unacceptably increase the morbidity [9] The prognosis is poor with chemotherapy being minimally effective in angiosarcomas with a modest (17 to 34%) response rate [3, 5]. Age, time of onset, depth of tumour invasion, and size of the clearance margin have been identified as possible associated factors; however, tumour grading was not related to the prognosis [3, 5].

Targeted therapy such as vascular endothelial growth factor (VEGF) inhibitors are being investigated as novel treatment options. This is because over-expression of VEGF is considered as a vital mechanism in tumour genesis [10]. A phase II study of 30 patients which analysed the efficacy of the VEGF inhibitors for locally advanced angiosarcoma and haemangioendothelioma has shown that only 17% of the patients had a partial response. However, 50% had stable disease with a mean time to progression of 26 weeks [10]. Several ongoing clinical trials are in progress for further evaluation [5].

Although radiation-induced angiosarcoma is described in literature, the development of bilateral angiosarcoma of the breast within a short interval following radiotherapy is extremely rare [11]. The incidence of developing bilateral breast angiosarcoma is extremely small: 0.25–2.6 per million women who underwent bilaterally breast conservative surgery with radiotherapy for invasive carcinoma [12]. Therefore, only a few case reports have been reported so far [12]. Furthermore, in our patient, a strong unknown genetic predisposition of radiation-induced tumours may have caused the described clinical picture as angiosarcomas developed quickly after radiotherapy and were very aggressive. This evidence shows that some angiosarcomas can occur in short time and is, thus, biologically different from angiosarcomas occurring 10–20 years following radiotherapy. However, this is an area that has not been studied in great detail yet mostly due to the rarity of the condition.

Although radiation-induced angiosarcoma of the breast is an aggressive tumour with poor prognosis, the benefits of radiation therapy for primary breast cancer clearly outweighs the overall risk of developing a sarcoma [3]. Therefore, in primary breast cancer treatment, adjuvant radiation following breast conservation surgery still remains the standard of care.

## 4. Conclusion

Radiation-induced angiosarcoma is a rare and aggressive disease which poses diagnostic and therapeutic challenges. The reported patient had an unusual presentation with a rapidly growing fungating breast mass with spontaneous bleeding and bilateral disease. Radical surgical excision with adjuvant chemotherapy is offered with potential for cure.
